# Study of Salidroside and Its Inflammation Targeting Emulsion Gel for Wound Repair

**DOI:** 10.3390/molecules28135151

**Published:** 2023-06-30

**Authors:** Xiaojie Wang, Jun Yang, Shuai Luo, Hucheng Zhang, Bo Liu, Zhiquan Pan

**Affiliations:** 1 School of Chemistry and Environmental Engineering, Wuhan Institute of Technology, Wuhan 430205, China; 100169@bpi.edu.cn; 2 School of Biological Engineering, Beijing Polytechnic, Beijing 100176, China; rainman@sina.com (J.Y.); luoshuai1985@sina.com (S.L.); zhanghchbj@163.com (H.Z.); 3 Britton Chance Center for Biomedical Photonics at Wuhan National Laboratory for Optoelectronics-Hubei Bioinformatics & Molecular Imaging Key Laboratory, Department of Biomedical Engineering, College of Life Science and Technology, Huazhong University of Science and Technology, Wuhan 430074, China

**Keywords:** salidroside, inflammation targeting, emulsion, gel, wound repair

## Abstract

Salidroside has been widely used in anti-tumor, cardiovascular, and cerebrovascular protection. However, there are few reports of its use for wound repair. Herein, salidroside inflammation-targeted emulsion gel and non-targeted emulsion gel were developed for wound repair. The inflammation-targeted emulsion gels showed an overall trend of better transdermal penetration and lower potential than non-targeted emulsion gels (−58.7 mV and −1.6 mV, respectively). The apparent improvement of the trauma surface was significant in each administration group. There was a significant difference in the rate of wound healing of the rats between each administration group and the model group at days 7 and 14. Pathological tissue sections showed that inflammatory cells in the epidermis, dermis, and basal layer were significantly reduced, and the granulation tissue was proliferated in the inflammation-targeted emulsion gel group and the non-targeted emulsion gel group. Regarding the expressions of EGF and bFGF, the expressions of bFGF and EGF in the tissues of the inflammation-targeted group at days 7, 14, or 21 were significantly higher than that of the non-targeted emulsion gel group and the model group, both of which were statistically significant compared with the model group (*p* < 0.05). These results demonstrated that salidroside has the potential as an alternative drug for wound repair.

## 1. Introduction

*Rhodiola rosea* comes from the genus Rhodiola in the family Rhodiidae, which is a commonly used herb in Tibetan medicine and is known as the “ginseng of the plateau” [1]. It is flat, sweet, and bitter and belongs to the lung and heart meridians, with the effects of benefiting vital energy, invigorating the blood, opening the veins, and calming asthma [2]. Salidroside is the phenolic secondary metabolism of *Rhodiola rosea*, which has a wide range of pharmacological effects, and related studies [3,4,5,6,7] mainly focus on enhancing human immunity, antioxidant, anti-aging, anti-tumor, cardiovascular and cerebrovascular protection, etc. Since 2011, it has been reported that salidroside has the effect of slowing down the inflammatory response [8], but the related studies are not deep enough. Molecular biology studies have shown that when inflammation occurs on the traumatic surface, the expression of cationic proteins such as transferrin is abnormally increased at the site of inflammation, which makes the inflammatory site positively charged [9]. At the same time, when skin damage and trauma are formed, a relatively stable current circuit is formed at the edge of the trauma [10]. If a negatively charged agent is given at this time, it can act on the damaged skin in time to excite the damaging endogenous electric field and accelerate the repair of the damaged skin through various changes in cellular mechanisms [11]. Therefore, combined with the pre-laboratory studies, we hypothesized that salidroside may play a role in promoting wound healing in the body by slowing down the inflammatory response and that negatively charged preparations that reach a certain potential value are beneficial to wound repair due to their better inflammatory targeting properties.

Emulsion gel is one of the common amphiphilic delivery systems, which is a gel with a gel network structure and solid-like mechanical properties formed by immobilizing internally emulsified lipid droplets in a hydrogel network [12]. Emulsion gels have been widely used in drug delivery and biomedical applications [13]. The emulsion is a biphasic formulation that consists of immiscible dispersed and dispersion phases stabilized via an emulsifier. Several types of emulsions, such as particle-stabilized oil/water and water/oil emulsions, have been fabricated for various applications in pharmaceuticals [14,15]. Emulsion gel systems have advantages over emulsions and hydrogels in their application. Compared with emulsions, emulsion gels can improve texture and dual controlled release properties as well as provide good thermodynamic stability. In addition, compared with pure hydrogels, emulsion gels are less dense due to the presence of lipids, which can reduce the frequency of administration and improve the bioavailability of the active substance. For example, Banerjee has developed natural gum (using a combination of an aqueous solution of xantham gum (X) and guar gum (G)) modified sunflower oil-based emulsion gels for the delivery of probiotic–drug combinations [16].

Herein, salidroside inflammation-targeting emulsion gels were developed for wound repair. We used salidroside inflammation-targeting emulsion gel and salidroside non-targeted emulsion gel to conduct wound-healing experiments in rats in vitro. The aim was to extend the therapeutic field of salidroside (promoting wound repair) and preliminarily investigate its mechanism of promoting wound repair, as well as to compare the therapeutic effects of salidroside inflammation-targeted emulsion gel with non-targeted emulsion gel on wound repair in rats.

## 2. Experimental Results

### 2.1. Preparation of Salidroside Inflammation Targeting Emulsion Gel

**Figure 1:**
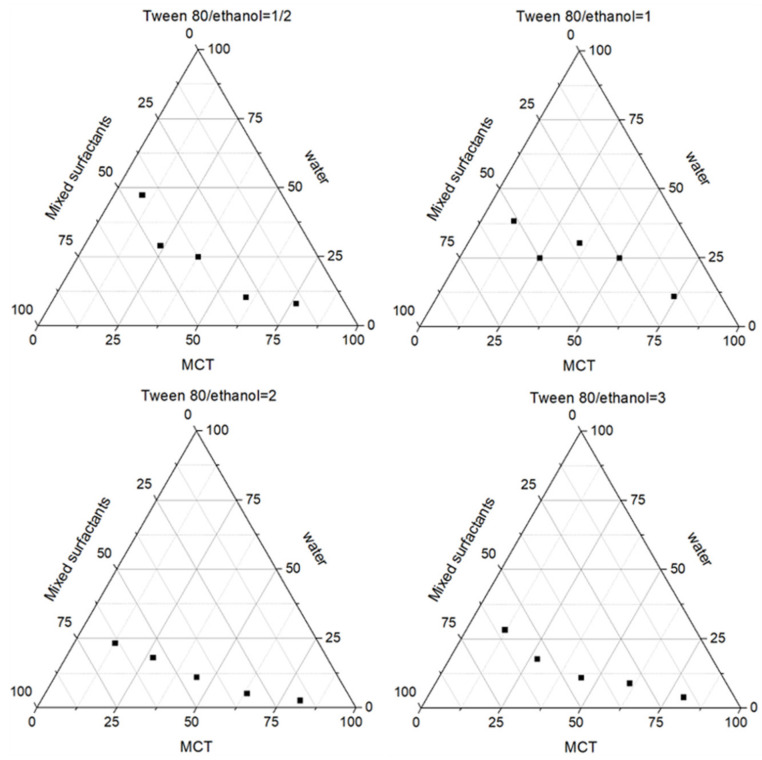
Pseudo-ternary phase diagrams of different ratios of the mixed surfactant and oil phase.

The mass ratio (Km value) of a surfactant and a co-surfactant can affect the formation of an emulsion [17]. To prepare stable salidroside inflammation targeting emulsion, MCT was used as an oil phase, and Tween 80 and anhydrous ethanol were used as a surfactant and a co-surfactant, respectively. The Km values were chosen to be 0.5, 1, 2, and 3. At room temperature, the surfactant was mixed with the co-surfactant at a certain Km value, and the mixed surfactant and oil phase were mixed according to the mass ratios of 1:5, 2:4, 3:3, 4:2, and 5:1 and titrated with the aqueous phase under a magnetic stirrer. The volume of the aqueous phase was recorded from clarification to turbidity, and the mass fraction of each component was calculated. The corresponding results are plotted as pseudo-ternary phase diagrams using the origin8.0 software. As shown in Figure 1, the area of the emulsion area was the largest when the Km value was 2, so the Km value was chosen as 2.

The potentials of the three batches of salidroside inflammation-targeted emulsions were measured as −58.3 mV, −59.1 mV, and −58.6 mV, respectively, with the mean value of −58.7 mV; the measured potentials of the three batches of non-targeted salidroside emulsions were −1.7 mV, −1.8 mV, and −1.3 mV, respectively, with the mean values of −1.6 mV.

### 2.2. Percutaneous Penetration

**Figure 2:**
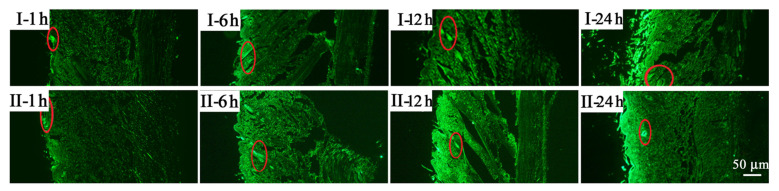
Fluorescence distribution of the skin after treatment with salidroside non-targeted emulsion gel (group I) and salidroside inflammation-targeting emulsion gel (group II) for 1, 6, 12, and 24 h. The red circles indicates the fluorescent signal. Scale bar is 50 μm.

The results of the transdermal fluorescence experiments showed that both the non-targeted salidroside emulsion gel and the targeted salidroside emulsion gel penetrated the skin tissue within 1 h after application. In most cases, the depth of penetration of the salidroside inflammation-targeted emulsion gel into the skin was slightly greater than that of the non-targeted salidroside emulsion gel (Figure 2). In addition, the fluorescence fraction was mainly distributed on the skin surface, hair follicles, and their appendages, indicating that the hair follicles and their appendages in the skin played a role in the transdermal absorption of the drug.

### 2.3. Morphological Changes in Rats

The morphological changes of wounds treated with different methods were shown in Table 1. After 7 days of administration, a few wounds were debrided. Group A had yellow exudate on the edge of the wound after the removal of the wound patches, and the whole wound was dark red. The wounds in group B had dark red patches with dry raised edges and no yellowish exudate and showed signs of healing. The undebrided wounds in groups C and D had no exudate, were dark red in color, hard in texture, with raised edges, and showed signs of healing, but the effect of group C was more pronounced than that of group D.

After 14 days of administration, the wounds in group A were red, with dry scabs and exudate. Group B was less effective, and there was no significant difference compared with group A. Groups C and D had smaller wound areas and pale red traumatic surfaces. The healed areas were smoother, lighter white in color, and less pigmented compared with the skin of group A. Of these groups, group C showed the most obvious improvement in healing.

After 21 days of administration, the wound surfaces of group A were bright red with obvious scarring. The wound surfaces of groups C and D were almost completely healed. In particular, wounds in group C had already grown hair.

### 2.4. Wound Healing Rate

Table 2 shows the effect of each group on the rate of wound healing in rats. Compared with the model group, an increase in the healing rate of wound surfaces was seen in the inflammation-targeted emulsion gel group and the non-targeted emulsion gel group after the 7 and 14 days of administration. However, no significant increase in wound healing rate was observed in rats at 21 days.

### 2.5. Pathological Section

**Figure 3:**
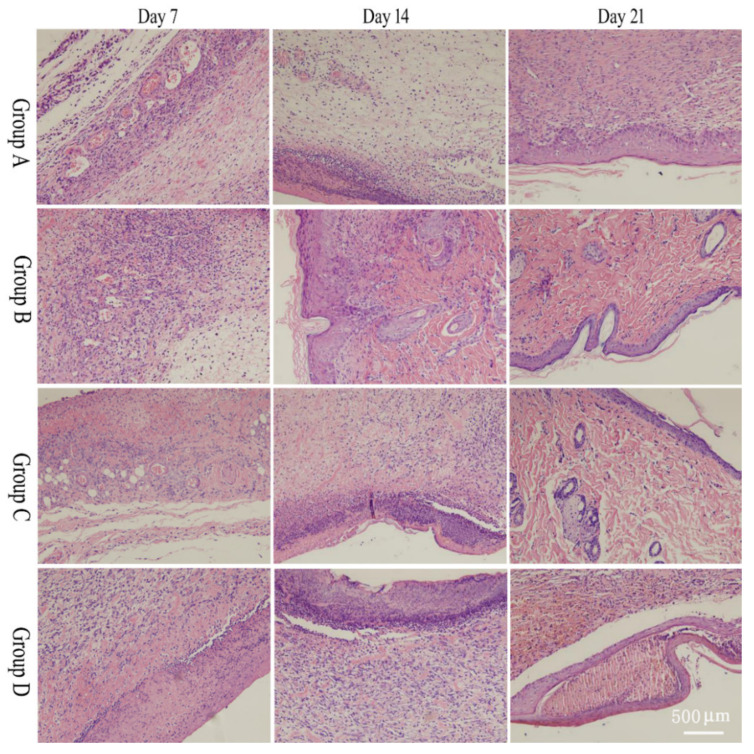
H&E staining result of wound tissue. Group A: model group, Group B: salidroside non-targeting emulsion gel group, Group C: salidroside inflammation-targeting emulsion gel group, Group D: positive group. Scale bar is 500 μm.

Four fields of view were selected to observe and image the morphological changes in rat wound tissue under a 10× microscope (Figure 3). After 7 days, the epidermal layer thickened in all groups. The skin of group A showed high infiltration of inflamed cells, but the treated groups showed less infiltration of inflamed cells. On day 14, tissues in group B tended to be evenly distributed, with hair follicles and sebaceous glands appearing. In groups C and D, inflammatory cell infiltration decreased, fibroblasts increased, and some hair follicles and sebaceous glands appeared, the most prominent of which were in group C. After 21 days, the epidermal structure of group A developed, with inflammatory cells still more infiltrated in the dermis and some fibroblasts visible. The epidermal tissue structure in group B neonates was relatively clear, with denser collagen fibers and few infiltrating inflammatory cells in the interstitium. In groups C and D, a complete epidermal layer was visible, and fresh hair follicle tissue was visible in the dermis, with hair follicles dense and visible. Group C was still the most obvious.

### 2.6. bFGF and EGF Expression

**Figure 4:**
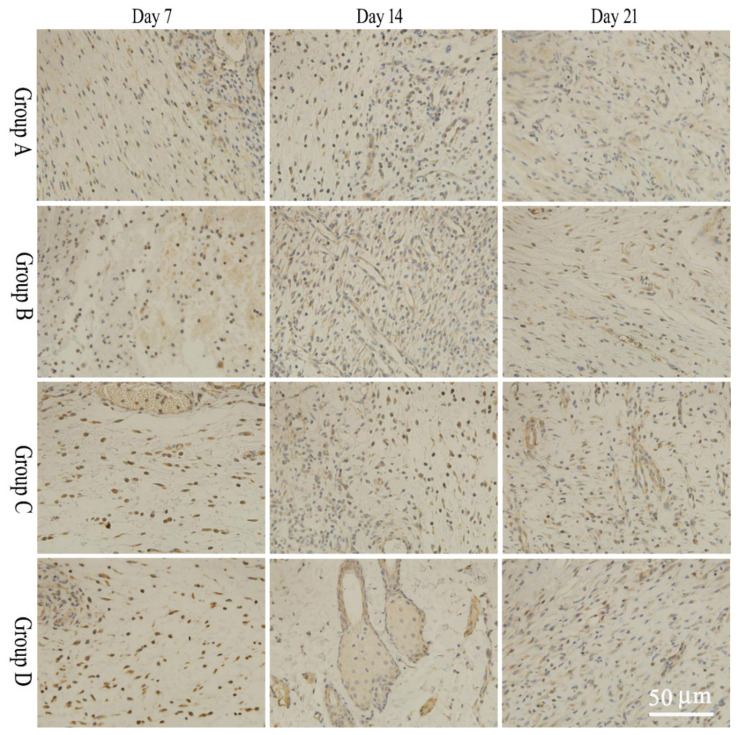
Expression results of bFGF at different time points. Group A: model group, Group B: salidroside non-targeting emulsion gel group, Group C: salidroside inflammation-targeting emulsion gel group, Group D: positive group. Scale bar is 50 μm.

**Figure 5:**
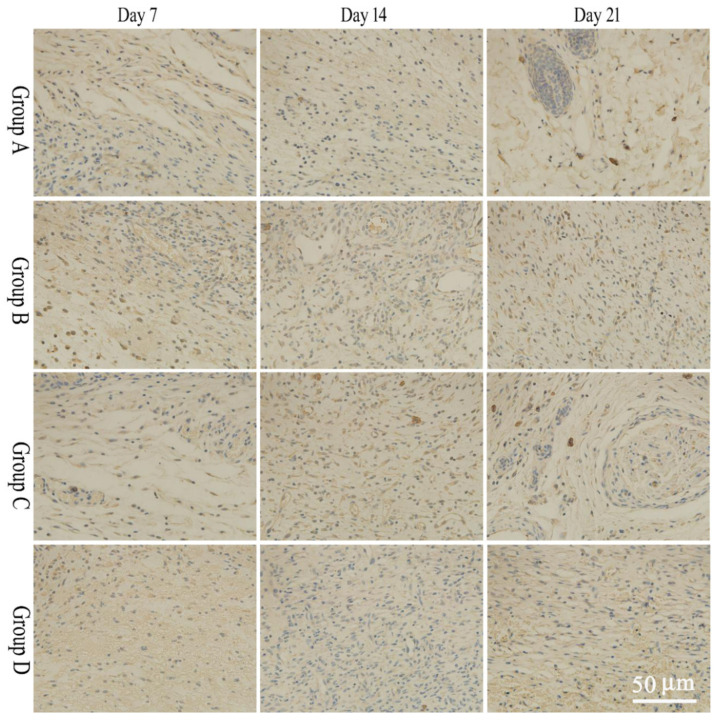
Expression results of EGF at different time points. A: model group, B: salidroside non-targeting emulsion gel group, C: salidroside inflammation-targeting emulsion gel group, D: positive group. Scale bar is 50 μm.

Four fields of view were selected for observation and imaging under a 40× microscope. IPP6.0 software was used to measure the average optical density of the whole tissue images. During the wound healing phase, bFGF expression in tissues in the salidroside inflammation-targeting emulsion gel group was significantly higher than in the non-targeted emulsion gel group and the control group on days 7 and 14, and the expression of EGF in the inflammation-targeted group was significantly higher than that of the non-targeted emulsion gel group and the model group on days 14 and 21. The results were statistically significant compared to the model group (*p* < 0.05). Specific results are shown in Figure 4 and Figure 5 and Table 3 and Table 4.

## 3. Materials and Methods

### 3.1. Animals

The total number of SD rats was half male and half female, and the body weight range was 200–220 g. The Certificate of Conformity No. was SCXK (Beijing, China) 2016-0002. They were purchased from Beijing SPF Laboratory Animal Technology Co. (Beijing, China) and housed in the animal house of Beijing University of Chinese Medicine.

### 3.2. Materials

Jingwanhong Ointment (Tianjin Darentang Pharmaceutical Jingwanhong Co., Ltd., Tianjin, China); 10% neutral formalin fixative (Beijing Yili Fine Chemicals Co., Ltd., Beijing, China); EGF antibody (Shanghai Yuanye Bio Co., Ltd., Shanghai, China); medium chain triglycerides (MCT) (Shanghai Yuanye Bio-Technology Co., Ltd., Shanghai, China); SDS (Shanghai Yuanye Bio-Technology Co., Ltd.); Tween 80 (Shanghai Yuanye Bio-Technology Co., Ltd.); bFGF antibody (Shanghai Yuanye Bio Co., Ltd.); Chloral hydrate (Tianjin Fuchen Chemical Reagent Factory, Tianjin, China); Sodium penicillin for injection (Jiangxi Keda Animal Pharmaceutical Co., Ltd., Fuzhou, China); Complex iodine disinfectant solution (Hunan Guangshengyuan Pharmaceutical Technology Co., Ltd., Changsha, China); Physiological saline (Jiangxi Keda Sanitary Products Co., Ltd., Fuzhou, China); Veet Silk & Fresh hair removal cream (Reckitt Benckiser China Co., Ltd., Beijing, China); Coumarin 6 reference product (Shanghai Yuanye Biotechnology Co., Ltd., Shanghai, China).

### 3.3. Instruments

Olympus BX51 microscope (OLYMPUS Co., Ltd., Tokyo, Japan); FA1204B analytical balance (Shanghai Precision Instruments Co., Ltd., Shanghai, China); Sartorius BS 110S electronic analytical balance (Beijing Sartorius Scientific Instruments Co., Ltd., Beijing, China); Electric thermostatic water bath (Tianjin Taiste Instrument Co., Ltd., Tianjin, China); 3–18 N desktop high-speed centrifuge (Hunan Hengnuo Instrument & Equipment Co., Ltd., Changsha, China); Ultrasonic cell crusher (Ningbo Xinzhi Technology Co., Ltd., Ningbo, China); X5 intelligent pet shaver (Handan Fengfeng Hanzhuo Trading Co., Ltd., Handan, China); KQ-3200DE CNC ultrasonic instrument (Kunshan Ultrasonic Instruments Co., Ltd., Kunshan, China); TS-2 fluorescence inverted biological microscope (Beijing Ruike Zhongyi Technology Co., Ltd., Beijing, China); Electronic balance (Shanghai Hengping Instrument Co., Ltd., Shanghai, China); Zetasize Nano ZS nanometer (Malvern, UK); Surgical instruments (gavage needles, syringes, tweezers, hemostatic forceps, gauze, cotton balls, cotton swabs, tissue scissors).

### 3.4. Preparation of Salidroside Inflammation Targeting Emulsion Gel

The inflammation-targeting emulsion was prepared via oily phase titrated with the aqueous phase. To obtain the oily phase, 1 g of Tween 80 and 0.5 mL of ethanol were mixed well and added 1.25 mL of MCT with magnetic stirring. To obtain the aqueous phase, 0.36 g of SDS was dissolved in 7.25 mL of pure water and poured into a centrifuge tube containing 10 mg of salidroside. Then, the aqueous phase was slowly added to the oil phase with magnetic stirring while adding until a homogeneous mixture was obtained. The inflammation-targeting emulsion can be obtained via ultrasound treatment for 5 min (2 s on and 2 s off). Additionally, 0.5 g carbomer and 15 g glycerol were ground well in a grinding vessel. PBS (90 mL) was added in and stirred well. The inflammation-targeting emulsion (10 mL) and carbomer gel were mixed uniformly to obtain the salidroside inflammation-targeting emulsion gel.

### 3.5. Preparation of Non-Targeted Salidroside Emulsion Gel

The preparation method was the same as 2.4, except that no charge conferring agent (SDS) was added.

### 3.6. Zeta Potential Measurement

An appropriate amount of emulsion was precisely pipetted and diluted 100 times with liquid paraffin. The emulsion dilution was aspirated using a 5 mL disposable syringe and slowly injected into the potentiometric beaker, carefully checked, and emptied of air bubbles. The potential was determined using a Zetasize Nano ZS nanometer.

### 3.7. In Vivo Fluorescence Transdermal Experiment

#### 3.7.1. Preparation of Fluorescently Labeled Emulsion Gel

Fluorescently labeled salidroside inflammation targeting emulsion gel and salidroside non-targeted emulsion gel were prepared by adding 100 μL of 0.5% coumarin 6 solution dropwise to the oil phase and then adding the aqueous phase according to the prescription process in Section 2.4.

#### 3.7.2. Preparation and Observation of Microscopic Samples

The dorsal skin of SD rats (*n* = 7) was prepared with a shaving device and hair removal cream and divided into two parts (each part was about 1 × 1 cm^2^). The fluorescently labeled salidroside inflammation-targeting emulsion gel (group I) and salidroside non-targeting emulsion gel (group II) were given with a fine glass rod. After 1, 6, 12, 24, and 48 h of anesthesia with chloral hydrate, each section of the skin was taken and fixed with 10% formaldehyde and then made into longitudinal ice-cut white slices via a series of operations such as dehydration, wax immersion, embedding and sectioning, which were frozen and stored away from light. The sections were placed under an inverted fluorescent microscope (40×) with an excitation wavelength of 488 nm for observation and image acquisition.

### 3.8. Preparation of Rat Wound Model and Experimental Grouping

Another group of rats was taken for adaptation to the laboratory environment for 3 d and then used for the experiment [18]. The rats were randomly divided into a model group (group A), a salidroside non-targeted emulsion gel group (group B), a salidroside inflammation-targeted emulsion gel group (group C), and a positive drug group (group D). The experiment was conducted on rats one by one. The backs of rats were dehaired with a shaver and hair removal cream and disinfected with alcohol wipe. Chloral hydrate (10%) was injected intraperitoneally for anesthesia. After the rats were anesthetized, the rats were placed laterally on the operating table. The skin of the back was lifted laterally, and two circular full-thickness skin excision wounds with a diameter of 18 mm were cut at 1 cm on each side of the left and right sides of the spine using surgical scissors, and the wound was disinfected promptly after the mapping was completed. Immediately after disinfection, pharmacological interventions were administered to different groups of rat models once daily. The model group was disinfected with iodine volt without any drugs, and each drug administration group was given different preparations with 0.2 g, which formed a 1 mm thick layer on the wound. After the operation, each rat was kept in a single cage, with a daily change in drugs and a free diet.

### 3.9. Wound Efficacy Indicators

#### 3.9.1. Gross Observation of Wounds

The rats were observed directly at the time of resuscitation after wound modeling, and the wound state was observed at 7, 14, and 21 days after wound modeling. Exudation, infection, crusting, and redness were recorded.

#### 3.9.2. Wound Healing Rates

On days 1, 7, 14, and 21 after wound reconstruction, the wound surfaces were traced with transparent sulfuric acid paper. The marked area on the paper was cut out, and the mass was weighed on a balance with an accuracy of 1/10,000 g. The healing rate was calculated by replacing the area with the mass and was presented from the following equation [19].

Wound healing rate = (Initial wound area-unhealed area at each time point)/Initial wound area

Using the area 1 day after the wound modeling as the initial area, the wound healing rates at 7, 14, and 21 days after wound modeling were calculated.

#### 3.9.3. Histopathological Observation

The wound pathology observation was performed using the H&E staining method, and the main steps include the following: removal, fixation, dehydration and transparency, immersion in wax, embedding, sectioning, patching, staining, and mounting. The general morphological structure of the skin tissue was observed under a microscope.

#### 3.9.4. Immunohistochemistry

At 7, 14, and 21 days after modeling, wound skin tissues of each group of rats were taken, and the changes of bFGF and EGF in the wound were assessed via immunohistochemistry. On the immunohistochemical sections, the positive cells were blue. The sections were placed under a 40× microscope, and positive protein expression was observed in each specimen selected in 4 fields of view. IPP-6.0 image analysis software was applied for image analysis, and the integrated optical density (IOD) values of the blue areas were calculated and averaged.

### 3.10. Statistical Analysis

All the data are presented as a mean value ± the standard deviation, and all experiments were repeated at least three times. The difference comparison between the two samples was calculated by *t* test.

## 4. Discussion

In this study, inflammation-targeted salidroside emulsion gels have more benefits in wound healing in rats than non-targeted salidroside emulsion gels. Its therapeutic effects mainly manifested via improving wound appearance, reducing inflammatory cell infiltration, and increasing fibroblast numbers. In addition, the tissue structure of the new epidermal layer is clearer, and collagen fibers are denser. The presumed reasons are the following:

Wound healing is a complex and dynamic biological process that involves four highly programmed phases: hemostasis, inflammation, proliferation, and remodeling. Among them, inflammation is the main step [20,21,22,23,24]. Wound formation leads to the continuous release of inflammatory mediators onto the wound. At the site of inflammation, the expression of cationic proteins (e.g., transferrin) is abnormally increased, making the site of inflammation positively charged [25,26,27]. Using this principle, Tirosh B [9] and Harel E [28] prepared negatively charged liposomes, Kesharwani SS [29] prepared Ora-Curcumin-S via molecular complexation of curcumin with a hydrophilic polymer Eudragit^®^ S100. They all achieved a better inflammation-targeting effect. Similarly, the inflammation-targeting emulsion gel used in the present work has a stronger negative charge than the non-targeting emulsion gel, which enables inflammation targeting.

The wound surface is equivalent to an endogenous electric field, and the targeted emulsion gel is equivalent to an exogenous electric field. The difference in microenvironment potential promotes cell movement around the injured tissue and accelerates healing. When the skin is damaged and a wound forms, the epithelial potential shortens, and a current flows out of the center of the wound, forming a relatively stable circuit at the edge of the wound [30,31,32]. When a negatively charged substance is applied directly to the wound, it is equivalent to the formation of a simple exogenous electric field, which causes many cells surrounding the damaged tissue to migrate in a specific direction under the applied electric field, thereby accelerating the recovery of the damaged tissue [10,11]. In this paper, the mean potential of the inflammation-targeted emulsion gel is −58.7 mV, and the mean potential of the non-targeted emulsion gel is −1.6 mV. The rate of migration of damaged tissue cells in specific directions under the influence of an electric field is positively related to the intensity of the electric field so that the time required for wound healing with an inflammation-targeted emulsion gel is relatively reduced.

Calculations show that there was a significant difference in the rate of wound healing of the rats between each administration group and the model group after the 7th and 14th day of administration; however, no significant increase in wound healing rate was observed in rats at 21 days. In the analysis, it is considered that the skin itself has the ability to heal and that the degree of healing is calculated simply by the area of wound repair, with the model group also having some degree of healing. However, the apparent observation clearly shows that the drug group has advantages over the control group and that the healing skin color and hair growth are more significant. The combination of H&E staining and immunohistochemistry results confirmed the benefits of the treatment group. Salidroside emulsion gel targeting inflammation has a superior effect.

The skin healing process is accomplished with epidermal cell division and proliferation, fibroblast proliferation, and extracellular matrix secretion. Various inflammatory cells and cytokines are involved in the wound healing process, among which EGF is the most important mitogen of epidermal cells, granulation tissue filling is also a key stage in wound healing, bFGF cells are the main functional cells in wound repair, i.e., EGF and bFGF are important cytokines involved in wound healing [33,34,35,36,37]. During the wound-healing process, the expression of bFGF and EGF increases. In this paper, we investigated the expression of bFGF and EGF on the wound surface. The expression of bFGF in the emulsion gel group with salidroside targeting inflammation was significantly increased after 7 d, 14 d, and 21 d of application compared with the model group (*p* < 0.05). The expression of EGF in the salidroside inflammation-targeting emulsion gel group after 14 d and 21 d of administration compared with the model group was significantly increased (*p* < 0.05). The expression of bFGF in the targeted emulsion gel group with inflammation was significantly increased (*p* < 0.05) compared with the non-targeted emulsion gel group after 7 d, and 14 d administration. The expression of EGF in the targeted emulsion gel group with inflammation was significantly increased (*p* < 0.05) compared with the non-targeted emulsion gel group after 14, and 21 d administration. This indicates that the charge-inflammation-targeted form of the formulation is more likely to promote the expression of EGF and bFGF in the wound tissue and facilitate the healing of the wound.

Currently, most semi-solid drug release experiments are carried out using vertical Franz diffusion chambers, and researchers often add surfactants or a certain volume fraction of lower alcohols to the receiving medium to achieve a better transdermal effect [38], which differs from physiological skin conditions. Although it is possible to obtain significant experimental results, it is difficult to account for the release of the drug in principle.

In this study, we used fluorescently labeled coumarin 6 formulations for in vivo fluorescent transdermal experiments in rats, which better simulate the actual physiological state of the skin. Compared to the diffusion chamber method, which is currently used to investigate the release rate of the semi-solid formulation, this method can represent the actual transdermal process of the emulsion gel after coating the skin surface, and the experimental results are more reliable. The results showed that the drugs in the inflammation-targeted emulsion gel group and the non-targeted emulsion gel group were able to penetrate the deeper layers of the skin and exert their therapeutic effect over time. Compared to the non-targeted emulsion gel group, the transdermal effect of the inflammation-targeted emulsion gel group was more pronounced, which may be related to the faster electrostatic adsorption between the inflammation-targeted emulsion gel and the positively charged inflamed tissue site.

## 5. Conclusions

In this study, we developed a salidroside inflammation targeting emulsion gel for wound repair. Compared with non-targeted emulsion gels, inflammation-targeted emulsion gels presented better transdermal penetration. The results of the wound-healing rate and pathological section showed that the wound treated with salidroside inflammation-targeting emulsion gel could heal faster and better compared with the salidroside non-targeted emulsion gel. In addition, bFGF expression in tissues in the salidroside inflammation-targeting emulsion gel group was significantly higher than in the non-targeted emulsion gel group and the control group on days 7 and 14. EGF expression in tissues in the salidroside inflammation-targeting emulsion gel group was significantly higher than in the non-targeted emulsion gel group and the control group on days 14 and 21.Therefore, this work opens up new research avenues for the development of wound-healing agents.

## Figures and Tables

**Table 1:** The morphological changes of wounds treated with different methods.

	7 d	14 d	21 d
Group A	Exudate, dark red wound	Exudate, red wound	No exudate, light red, hairless growth
Group B	No exudate, dark red wound	Exudate, red wound	No exudate, light red, hairless growth
Group C	No exudate, dark red wound	No exudate, light red wound; smooth and light white healing area with little pigmentation	No exudate, light red, hairy growth
Group D	No exudate, dark red wound	No exudate, light red wound; smooth and light white healing area	No exudate, light red, hairless growth

A: model group, B: salidroside non-targeting emulsion gel group, C: salidroside inflammation-targeting emulsion gel group, D: positive group.

**Table 2:** Comparison of wound healing rate in different time groups (x ± s).

Group	Wound Healing Rate		
	Day 7	Day 14	Day 21
Group A	0.50 ± 0.09	0.71 ± 0.17	0.83 ± 0.23
Group B	0.57 ± 0.10	0.76 ± 0.19	0.84 ± 0.19
Group C	0.68 ± 0.12 *	0.83 ± 0.21 *	0.87 ± 0.20
Group D	0.64 ± 0.13	0.82 ± 0.20	0.86 ± 0.21

A: model group, B: salidroside non-targeting emulsion gel group, C: salidroside inflammation-targeting emulsion gel group, D: positive group. * *p* < 0.05 compared with the model group.

**Table 3:** Expression of bFGF in granulation tissue of rats at different stages (x ± s).

Group	Expression of bFGF in Granulation Tissue (pg/mL)		
	Day 7	Day 14	Day 21
Group A	7.14 ± 1.23	10.21 ± 2.36	7.41 ± 1.91
Group B	11.53 ± 2.67	11.79 ± 2.55	10.00 ± 2.03
Group C	16.87 ± 2.42 *^,∇^	16.63 ± 2.57 *^,∇^	15.68 ± 4.52 *
Group D	19.53 ± 8.37 *^,∇^	11.36 ± 2.37	12.96 ± 3.31 *

Note: Compared with the model group, * *p <* 0.05; compared with the non-targeted emulsion gel group, ^∇^ *p <* 0.05.

**Table 4:** Expression of EGF in granulation tissue of rats at different stages (x ± s).

Group	Expression of EGF in Granulation Tissue (pg/mL)		
	Day 7	Day 14	Day 21
Group A	6.32 ± 1.06	4.49 ± 0.86	7.21 ± 0.88
Group B	6.42 ± 1.34	6.08 ± 0.58	7.91 ± 1.88
Group C	6.43 ± 1.76	11.62 ± 3.04 *^,∇^	18.86 ± 0.94 *^,∇^
Group D	9.47 ± 2.46	8.67 ± 2.15 *	11.03 ± 3.06 *

Note: Compared with the model group, * *p <* 0.05; compared with the emulsion gel group, ^∇^ *p <* 0.05.

## Data Availability

Not applicable.

## Sample Availability

Samples of the compounds are not available from the authors.
